# Co-production of DHA and squalene by thraustochytrid from forest biomass

**DOI:** 10.1038/s41598-020-58728-7

**Published:** 2020-02-06

**Authors:** Alok Patel, Stephan Liefeldt, Ulrika Rova, Paul Christakopoulos, Leonidas Matsakas

**Affiliations:** Biochemical Process Engineering, Division of Chemical Engineering, Department of Civil, Environmental, and Natural Resources Engineering, Luleå University of Technology, SE-971 87, Luleå, Sweden

**Keywords:** Industrial microbiology, Applied microbiology

## Abstract

Omega-3 fatty acids, and specifically docosahexaenoic acid (DHA), are important and essential nutrients for human health. Thraustochytrids are recognised as commercial strains for nutraceuticals production, they are group of marine oleaginous microorganisms capable of co-synthesis of DHA and other valuable carotenoids in their cellular compartment. The present study sought to optimize DHA and squalene production by the thraustochytrid *Schizochytrium limacinum* SR21. The highest biomass yield (0.46 g/g_substrate_) and lipid productivity (0.239 g/g_substrate_) were observed with 60 g/L of glucose, following cultivation in a bioreactor, with the DHA content to be 67.76% w/w_total lipids_. To reduce costs, cheaper feedstocks and simultaneous production of various value-added products for pharmaceutical or energy use should be attempted. To this end, we replaced pure glucose with organosolv-pretreated spruce hydrolysate and assessed the simultaneous production of DHA and squalene from *S. limacinum* SR21. After the 72 h of cultivation period in bioreactor, the maximum DHA content was observed to 66.72% w/w_total lipids_ that was corresponded to 10.15 g/L of DHA concentration. While the highest DHA productivity was 3.38 ± 0.27 g/L/d and squalene reached a total of 933.72 ± 6.53 mg/L (16.34 ± 1.81 mg/g_CDW_). In summary, we show that the co-production of DHA and squalene makes *S. limacinum* SR21 appropriate strain for commercial-scale production of nutraceuticals.

## Introduction

Omega-6 (n-6) and omega-3 (n-3) are polyunsaturated fatty acids (PUFAs), whose precursors include linoleic acid and alpha-linolenic acid. They are considered essential because humans cannot synthesize them due to insufficient levels of elongases and delta-6-desaturases^1,2^. Although conversion of alpha-linolenic acid into omega-3 fatty acids, such as eicosapentaenoic acid (C_20:5n−3_), docosapentaenoic acid (C_22:5n−3_), and docosahexaenoic acid (C_22:6n−3_, DHA) occurs in humans, it happens at a very slow rate^3^ and so these fatty acids must be provided by the diet. These PUFAs have several health benefits and have been found to be very effective in the preventions and treatment of fetal diseases^4^. DHA plays a major role in cell signalling and is found mainly in the brain and retina tissues^5^. Additionally, it acts as an anti-inflammatory agent, a precursor of several metabolites and potent lipid mediators, and could be used to treat several cardiovascular or neurologic disorders, such as hypertension and Alzheimer’s^6^. Fish of the Salmonidae, Scombridae, and Clupeidae families have been the sole commercial source of DHA as their oil contains approximately 20 to 30% DHA^3^. However, DHA purification and concentration from fish oil is costly and the resulting oil quality depends on species, location, and pollution^7^. Furthermore, fish-derived DHA is unsuitable for vegetarians, and diminishing fish stocks together with marine pollution limit the increasing demand for DHA^7^. Oils from genetically engineered plant oilseeds, such as *Brassica juncea, Arabidopsis thaliana*, and *Camelina sativa* can, to some extent, fulfil the demand for DHA; however, omega-3 PUFAs content in vegetable oils is very low and there is no method to concentrate them for commercial purposes^8^. The production of plant oil is totally dependent on the environmental factors and the accessibility of arable land. In comparison, oleaginous microorganisms achieve better productivity of omega-3 fatty acids than plants^9^; therefore, microalgae, fungi, and bacteria could serve as a renewable and sustainable source of DHA.

Microalgae are natural producers of omega-3 PUFAs and can be grown under autotrophic, mixotrophic, and heterotrophic conditions^10^. The unicellular structure of microalgae facilitates the cultivation at large scale contrast to fungi where they form mycelial structure^11^. Thraustochytrids represent a marine group of oleaginous eukaryotic protists (class Labyrinthula of the Chromista Kingdom) often referred to as microalgae, even though they lack photosynthetic capability^12^. Many species of the genera *Schizochytrium*, *Thraustochytrium*, and *Ulkeniain* grow exclusively heterotrophically and accumulate considerable amounts of triacylglycerols with a high proportion of long-chain PUFAs, particularly DHA, which makes them suitable for commercial exploitation^13^.

*Schizochytrium limacinum* SR21, also known as *Aurantiochytrium limacinum* SR21, was selected for the present study. Isolation, identification, and optimization of cultivation conditions in flasks to enhance DHA production have been described before; however, few have offered insights into bioprocess development potential and the use of sources other than pure glucose or glycerol. Although crude glycerol has been tested on *S. limacinum* SR21, the presence of impurities (e.g., methanol and soap) can inhibit growth and lower DHA productivity. The commercial viability of microbial oils must be supported by inexpensive raw materials. Here, forest biomass consisting of non-edible lignocellulosic feedstocks was used. Forest-based industries contribute substantially to the Swedish economy and rely on vast swaths of forests, particularly those of Norway spruce (*Picea abies*), which accounts for 40.8% of the forest’s total standing volume^14^. Production of high-value nutraceutical compounds from side- and waste-streams of the forest sector could offer an important additional source of revenue to the forest-based industry. At the same time, nutraceuticals-based industries need to make upstream and downstream processing of DHA more sustainable and more economical^13^ by, for example, integrating it with simultaneous production of various value-added products for the pharmaceutical and petrochemical industries. Most thraustochytrids produce several antioxidants, such as β-carotene, squalene, and astaxanthin^15^. *Aurantiochytrium*/*Schizochytrium* species produce the polyunsaturated hydrocarbon triterpenoid squalene (2,6,10,15,19,23-hexamethyl-6,6,10,14,18,20-tetracosahexane)^16^. The most common source of squalene is liver oil of deep-sea sharks and whales; however, contamination with heavy metals and polychlorinated biphenyls poses a safety risk, while the oil’s often putrid odor and unpleasant taste diminish its appeal to consumers^17^. Furthermore, downstream processing of squalene from liver oils is hampered by the presence of chemically similar compounds such as cholesterol. Squalene can reduce serum cholesterol levels, enhance the immune response, and suppress tumor proliferation^18,19^, as well as increase immune responsiveness to vaccines^20^. In cosmetics, it is used as an antioxidant to quench singlet oxygen (^1^O_2_)^21^. Recently its value towards the production of high-grade aviation fuels has gained interest. Squalene is branched hydrocarbon that can be converted into smaller alkanes by catalytic conversion using ruthenium on ceria (Ru/CeO_2_) without skeletal isomerization and aromatization^22^. It has been already mentioned that Ru/CeO2-alcohol thermal catalysts are significantly superior to conventional methods using metal‐acid bifunctional catalyst^23^. Squalene obtained from *Botryococcus braunii* was transformed into bio jet fuel by using Ru/CeO2-alcohol thermal catalyst^23^_._ Due to increasing demand of squalene it cannot be fulfilled by mining from liver of marine mammals which is even not sustainable option for aquatic ecosystem^24^. Some plants can synthesize squalene but the quantities is not sufficient for commercial usage^25^. The only prominent source of squalene is thraustochytrids that can cultivate on industrial scale^26^.

In the present study, DHA and squalene co-production from *S. limacinum* SR21 were optimized in flask and bioreactor at batch conditions. Heterotrophic cultivation of *S. limacinum* SR21 was carried out on saccharified organosolv-pretreated forest biomass, to test a cost-effective and sustainable production of microbial DHA.

## Results and Discussion

### Batch cultivation of *S. limacinum* SR21 in Erlenmeyer flasks

**Figure 1:**
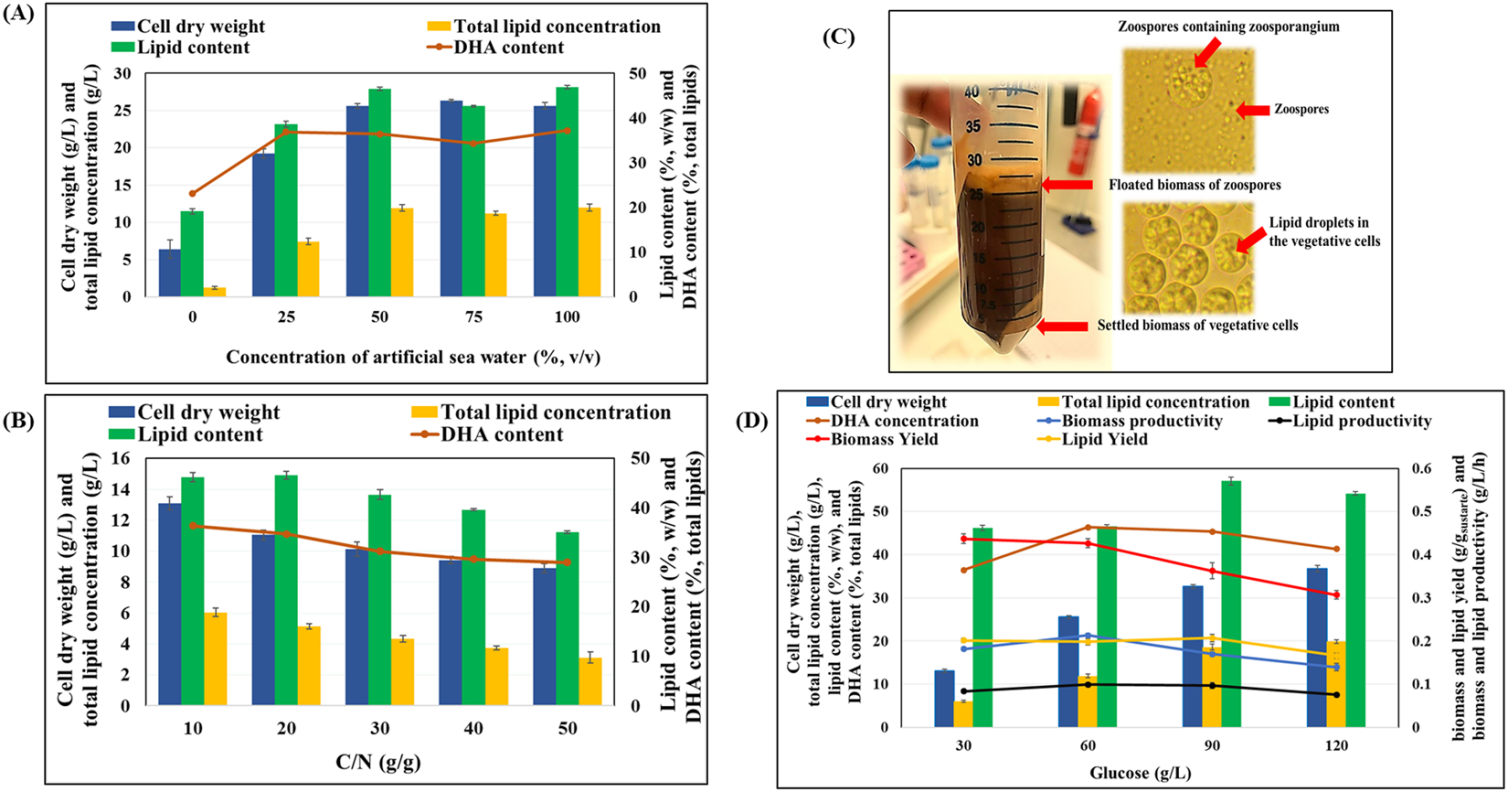
**(A)** Cultivation of *S. limacinum* SR21 at various concentrations of artificial seawater with fixed 30 g/L of glucose and C/N (g/g) ratio of 10. **(B)** Effect of various C/N (g/g) ratios on cell dry weight (g/L), total lipid concentration, lipid content and DHA content of *S. limacinum* SR21 cultivated in Erlenmeyer flasks. Growth medium contained 30 g/L of glucose and 50% of artificial seawater. **(C)** Cells of *S. limacinum* SR21 cultivated at a C/N ratio of 20 and with 30 g/L glucose were separated into two layers after centrifugation at 8000 rpm (7881*g) for 10 min. **(D)** Effect of various concentrations of glucose on cell dry weight (g/L), total lipid concentration (g/L), lipid content (%. w/w), DHA content (%, total lipids), biomass and lipid yield (g/g substrate), and biomass and lipid productivity (g/L/day) of *S. limacinum* SR21 cultivated in flasks. Data values represent average with standard deviation.

Initially, we aimed to enhance total lipid concentration and increase DHA content by first optimizing the concentration of artificial seawater in growth medium during batch cultivation of *S. limacinum* SR21 in Erlenmeyer flasks. This was done by varying the amount of artificial seawater from 25% to 100% (v/v) at a fixed concentration of glucose (30 g/L) along with a C/N ratio of 10 adjusted by yeast extract (Fig. 1A). While this strain can survive well at 0% artificial seawater, cell dry weight (6.43 ± 1.23 g/L) and lipid concentration (1.23 ± 0.21 g/L) were low under these conditions. The highest cell dry weight (13.1 ± 0.43 g/L) and total lipids (6.05 ± 0.28 g/L or 46.18 ± 0.92 w/w of lipid content) were observed with 50% artificial seawater (Fig. 1A). The DHA content was reported the lowest (23.12% of total lipid) at 0% artificial seawater, while 36.87% DHA was observed at 50% artificial seawater. The DHA content was not significantly increased with increasing concentration of artificial seawater from 50 to 100% (Fig. 1A). Kim *et al*. (2015) suggested that increasing the concentration of sea salt from 2 g/L to 20 g/L reduced cell rupturing of *Aurantiochytrium* sp. KRS101 and augmented biomass from 13.61 to 17.31 g/L^27^.

Limitation of key nutrients, such as nitrogen and phosphorus, causes stress to oleaginous microorganisms, retards growth, and leads to the conversion of excess carbon sources into lipids^28^. To study the effect of varying the C/N (g/g) ratio from 10 to 50, *S. limacinum* SR21 was cultivated at a fixed glucose concentration of 30 g/L and 50% artificial seawater (Fig. 1B). The highest cell dry weight (13.1 ± 0.43 g/L) and total lipid concentration (6.05 ± 0.28 g/L), corresponding to 46.18 ± 0.92% w/w lipid content, were obtained at C/N 10. At a C/N ratio of 20, lipid content remained similar (46.61 ± 0.81% w/w), but cell dry weight and lipid accumulation were only 11.05 ± 0.31 g/L and 5.15 ± 0.16 g/L, respectively. Increasing the C/N ratio to 30, 40, and 50 resulted in a further decrease of biomass and lipid formation (Fig. 1B). The DHA content was also decreased from 36.43% to 28.98% with increasing C/N ratio from 10 to 50 (Fig. 1B). Although higher C/N ratios are reportedly beneficial for high lipid accumulation in oleaginous microorganisms, this was not our case. The decreased biomass and lipid synthesis were likely due to lower levels of yeast extract, and consequently fewer vitamins and minerals, at increasing C/N ratios. Huang *et al*.^29^ showed that increasing the C/N ratio from 1.25 to 1.875 (with 75 g/L glycerol) promoted lipid accumulation; however, the accompanying reduction in biomass severely decreased total lipid and DHA concentrations^29^.

Interestingly, at a high C/N ratio, growth shifted from a vegetative to a reproductive phase, resulting in lower biomass and lipids. At a low C/N ratio, all glucose was consumed before reaching stationary phase; while at a high C/N ratio, the cells started to form zoosporangia and released zoospores into the medium (Fig. 1C), before glucose was consumed. A representative microscopic image of *S. limacinum* SR21 cultivated on 60 g/L of glucose with two different C/N ratio (10 and 50) is presented in Supplementary Fig. S1, where it is showing the formation of zoosporangia as a result of sexual reproduction at high C/N ratio and the emptied cells are clearly visualized after releasing of zoospores from the cells. Abad and Turan^30^ reported that discharge of zoospores from *A. limacinum* occurred only at the highest growth rate^30^. *Aurantiochytrium mangrovei* MP2 and *Aurantiochytrium* sp. KRS101 are susceptible to cell rupturing in freshwater medium, causing their cytoplasm to be released into the medium in a phenomenon that is highly dependent on nutrients’ availability^27,31^. Kim *et al*.^27^ suggested that cell rupture was more evident at lower C and N concentrations^27^. Whereas these authors observed a floating layer of small lipid bodies after centrifuging the cultures^27,31^, we could not dissolve the floating layer in chloroform and methanol (2:1 v/v) solution, ruling out the presence of lipid droplets. We believe that the floating layer could be explained by the shift from vegetative to zoosporic phase, whereby cells burst and release zoospores in the medium. Recently, evidence of sexual reproduction and morphological characterization of vegetative cells as well as zoosporangia of *Aurantiochytrium acetophilum*, *A. limacinum*, and *Schizochytrium mangrovei* was described^32^.

Once artificial seawater content and C/N ratio were optimized, different concentrations of glucose were tested at a range of 30–120 g/L (Fig. 1D). Cell dry weight increased from 13.1 ± 0.36 to 25.59 ± 0.32, 32.64 ± 0.47, and 36.78 ± 0.76 g/L as glucose increased from 30 to 60, 90, and 120 g/L of glucose. The corresponding lipid concentrations were 6.05 ± 0.24, 11.91 ± 0.43, 18.62 ± 0.72, and 19.91 ± 0.43 g/L. The increments in biomass and total lipid concentration were significant when shifting cultivation from 30 to 60 g/L but not when going from 90 to 120 g/L of glucose. The highest lipid content of 57.05 ± 0.89% w/w was recorded with 90 g/L of glucose, whereas the highest biomass productivity (0.21 g/L/h) and lipid productivity (0.09 g/L/h) were observed with 60 g/L of glucose (Fig. 1D). The DHA content in the total lipids was 36.43% with 30 g/L glucose and increased to 45.54% with 60 g/L glucose. Further increasing glucose concentration didn’t show any significant increment in DHA content (Fig. 1D). Chi *et al*.^33^ cultivated *S. limacinum* SR21 (ATCC MYA-1381) on glucose. They reported cell dry weight and biomass productivity of 18.47 g/L and 3.08 g/L/day (0.12 g/L/h), respectively when growing cells on 90 g/L of glucose^33^. Thus, biomass was higher in the present study, which could be attributed to complete glucose utilization. In a different study, whereby *S. limacinum* SR21 was cultivated on 30 to 120 g/L of glucose, biomass increased continuously up to 90 g/L of glucose and decreased thereafter; notably, cell dry weight (24.2 g/L) and lipid concentration (18.2 g/L) were also highest at 90 g/L of glucose^34^. These cell dry weight and biomass productivity values were still lower than those observed in the present study with 60 g/L of glucose, which may be explained by a different amount of yeast extract and cultivation conditions. Similar substrate inhibition at higher C source concentration was observed by Yokochi *et al*.^35^ when *S. limacinum* SR21 was cultivated with 30 to 120 g/L of glucose. Biomass synthesis became inhibited when the initial glucose concentration reached > 90 g/L^35^.

### Optimization of aeration rate and glucose concentration during batch cultivation in a bioreactor

**Figure 2:**
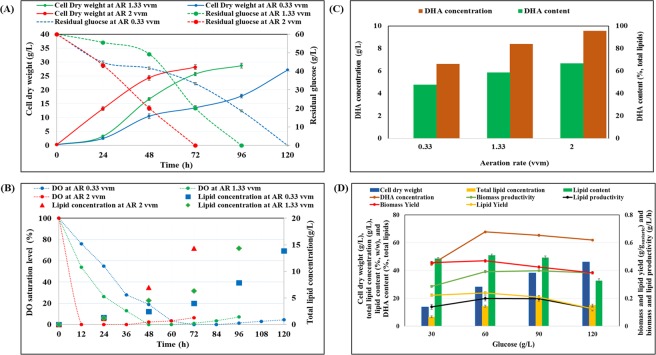
Effect of various aeration rates on **(A)** cell dry weight and glucose utilization, as well as **(B)** DO (%) and lipid concentration of *S. limacinum* SR21 cultivated on 60 g/L of glucose in a bioreactor. **(C**) Effect of aeration rate on DHA content and concentration of *S. limacinum* SR21 cultivated on 60 g/L of glucose in a bioreactor **(D)** Effect of glucose concentration on cell dry weight (g/L), total lipid concentration (g/L), lipid content (% w/w), biomass and lipid yield (g/g substrate), and biomass and lipid productivity (g/L/h) of *S. limacinum* SR21 cultivated in a bioreactor. Data values represent average with standard deviation.

Oxygen plays a crucial role in biomass, lipid, and DHA production^36^. Once the C/N ratio and concentration of artificial seawater were optimized, aeration rate was assessed by testing different flow rates (0.66, 1.33, 2, 2.66 vvm) in a 3-L bioreactor with 1 L working volume (Fig. 2A). Glucose utilization was severely affected by different aeration rates: at 0.66 vvm, glucose was fully consumed after 120 h of cultivation; whereas at 2 vvm, glucose was consumed in less than 72 h (Fig. 2A). Although biomass concentration was similar (~28 g/L) in all cases, the significantly different glucose consumption rates affected biomass productivity (g/L/h). At 2 vvm, cells grew faster than at 1.33 or 0.66 vvm, which caused utilization of all available oxygen in the medium. A high aeration rate in the initial phase of growth was responsible for high cell density; whereas later on, low oxygen promoted lipid synthesis (Fig. 2B). At high cell concentrations, oxygen consumption may exceed its supply, leading to oxygen limitation^37^. Surprisingly, at a high aeration rate (2 vvm), DO rapidly dropped to zero after 12 h and remained < 10% of saturation until 72 h of cultivation (Fig. 2B). Biomass synthesis reached 13.24 g/L at 24 h, which was almost 4 times higher than the value obtained with 1.33 vvm and at this stage lipid accumulation remained very low (only 1.11 g/L) (Fig. 2A,B). No significant changes were observed in biomass or lipid accumulation and glucose consumption when the aeration rate was shifted from 2 to 2.66 vvm (data not shown), hence 2 vvm was selected for further experiments. A high aeration rate has been reported to increase cell growth rate^38,39^, which supports our results. The DHA content (%, total lipids) was 47.76%, 58.65% and 66.72% at 0.33, 1.33 and 2 vvm of aeration rate, respectively (Fig. 2C). The abundance of DO in cultivation medium promotes cell respiration, energy metabolism, and carbon flux to the tricarboxylic acid cycle, eventually favoring high growth rate and production of valuable metabolites^39^. There are two contradictory reports: one suggests that molecular oxygen is required for desaturation during PUFA synthesis in most oleaginous microorganisms^40^; the other claims that DHA production can be enhanced by low DO saturation level^41^. A possible explanation for these two hypotheses is the existence of two distinct pathways for the biosynthesis of DHA in thraustochytrids^42^. The first occurs via an oxygen-dependent desaturation/elongation pathway (fatty acid synthesis pathway; FAS), in which molecular oxygen is required to start the synthesis of 18:4–6,9,12,15 from 18:3–9,12,15 by Δ6 desaturase, and elongation to 20:4–8,11,14,17^43,44^. The other, oxygen-independent polyketide synthase pathway (PKS) only occurs in bacteria and some eukaryotes including *Schizochytrium*^42^. Chang *et al*. (2013) showed that a continuous supply of oxygen to a culture of *Schizochytrium* sp. S31 enhanced growth and DHA synthesis, whereas a high oxygen transfer rate enhanced metabolic activity and promoted substrate assimilation to produce acetyl-CoA and NADPH for lipid synthesis^45^, confirming an earlier report^40^. Based on the genome annotation results of *S. limacinum* SR21, Ye *et al*., (2015) suggested that this microorganism doesn’t have delta-4 desaturase and some ORFs were considered to be as PKS proteins. These results confirm that the DHA could not be synthesized by FAS route but rather utilizes PKS pathway for DHA synthesis^46^. However, similar to fatty acids synthesis pathway, it requires a high amount of acetyl-CoA and NADPH for DHA synthesis via PKS pathway^42,46–48^.

After optimizing the aeration rate, we identified the highest applicable glucose concentration (Fig. 2D). Cell dry weight increased continuously from 13.69 ± 0.65 to 46.03 ± 0.69 g/L as glucose increased from 30 to 120 g/L. Similarly, lipid concentration increased from 6.65 ± 0.54 to 18.81 ± 0.73 g/L when glucose increased from 30 to 90 g/L; but dropped to 15.03 ± 0.58 at 120 g/L of glucose. The highest lipid content (50.91% w/w), lipid productivity (0.20 g/L/h), biomass yield (0.47 g/g_substrate_), and lipid yield (0.24 g/g_substrate_) were observed at 60 g/L of glucose. Similarly, when Chen and Yang (2018) cultivated *Thraustochytrium* sp. BM2 at glucose concentrations ranging from 15 to 90 g/L, the highest lipid content with maximum DHA productivity was observed at 60 g/L of glucose, however, the highest biomass productivity was reported at 75 g/L of glucose but dropped thereafter^49^. In flasks, both cell dry weight and lipids increased continuously with the consumption of glucose, irrespective of its concentration. Instead, in a bioreactor, biomass synthesis was rapid in the early log phase, after which glucose was consumed to support lipid synthesis, as demonstrated by lipid-free biomass. The different levels of biomass and lipid production exhibited by thraustochytrid strains in flask compared to bioreactor cultivation have not been explained yet. To investigate the effect of glucose utilization by *S. limacinum* SR21 on cell dry weight, lipid concentration, and lipid content, time-course experiments were performed in flasks and a bioreactor (Supplementary Fig. S2). Iida *et al*.^50^ suggested that *Thraustochytrium roseum* grew better in flasks because high mechanical stirring inhibited growth in the bioreactor^50^. In contrast, Nakahara *et al*. suggested the opposite was true for *Schizochytrium* sp. SR21 because the cells of this strain is extremely resistant to mechanical stirring^51^.

### Fatty acid profile and DHA content in *S. limacinum* SR21 lipids

**Figure 3:**
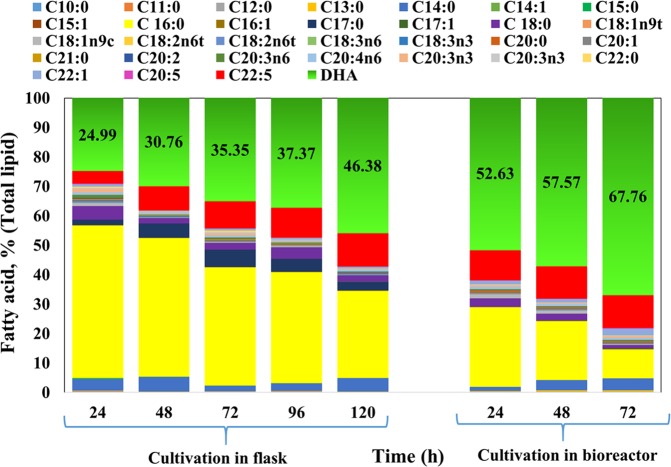
Fatty acid profile (% w/w_total lipid_) of *S. limacinum* SR21cultivated on an optimized concentration of glucose (60 g/L) in Erlenmeyer flasks and a bioreactor.

The fatty acid profile of *S. limacinum* SR21 cultivated in 60 g/L of glucose is presented in Fig. 3. All thraustochytrid strains have potential to synthesize mainly myristic acid (C14:0), palmitic acid (C16:0), stearic acid (C18:0), docosapentaenoic acid (C22:5) and docosahexaenoic acid C22:6 (DHA) whereas the composition and ratio usually varies with cultivation time^52^. After 24 h, the fatty acid profile from flask cultivation was C_14:0_ (3.9%), C_15:0_ (0.22%), C_16:0_ (51.77%), C_17:0_ (1.77%), C_18:0_ (4.59%), C_22:5_ (4.28%) and DHA (24.99%). In a bioreactor, the values doubled for DHA (53.63%). In flask cultivation, DHA content increased constantly from 24.99% at 24 h to 46.37% at 120 h (stationary phase); whereas, in a bioreactor, DHA content increased from 53.63% at 24 h to 66.72% at 72 h. The increment in DHA content in the course of cultivation might be related to declining DO in the medium. Due to increased utilization of carbon source with high aeration rate in the bioreactor, synthesis of biomass and lipid accumulation along with DHA content was significantly higher than those reported with flask cultivation. It has been reported previously that the high oxygen supply in the initial stage of cultivation leads to high substrate utilization capacity that further increase the lipid synthesis^53^.

Instead, Chang *et al*. (2013a) proposed that *Schizochytrium* sp. S3 produced more DHA in a baffled flask than in an unbaffled flask because the mass transfer coefficient (kLa) of oxygen was higher^45^. DHA synthesis in thraustochytrid species is thought to occur via two routes: the aerobic fatty acid synthase pathway, whereby oxygen is required in a series of desaturation and elongation steps^54^; and the oxygen-independent polyketide synthases pathway, whereby dehydration and isomerization reactions involving fatty acyl intermediates elongate the carbon chain without requiring dissolved oxygen (DO) during fermentation in later growth stages^55^. Chi *et al*. (2009) suggested that DHA production in *S. limacinum* SR21 could be improved by shifting DO levels during cultivation^56^.

### Growth and lipid production by *S. limacinum* SR21 cultivated on OPSH

**Figure 4:**
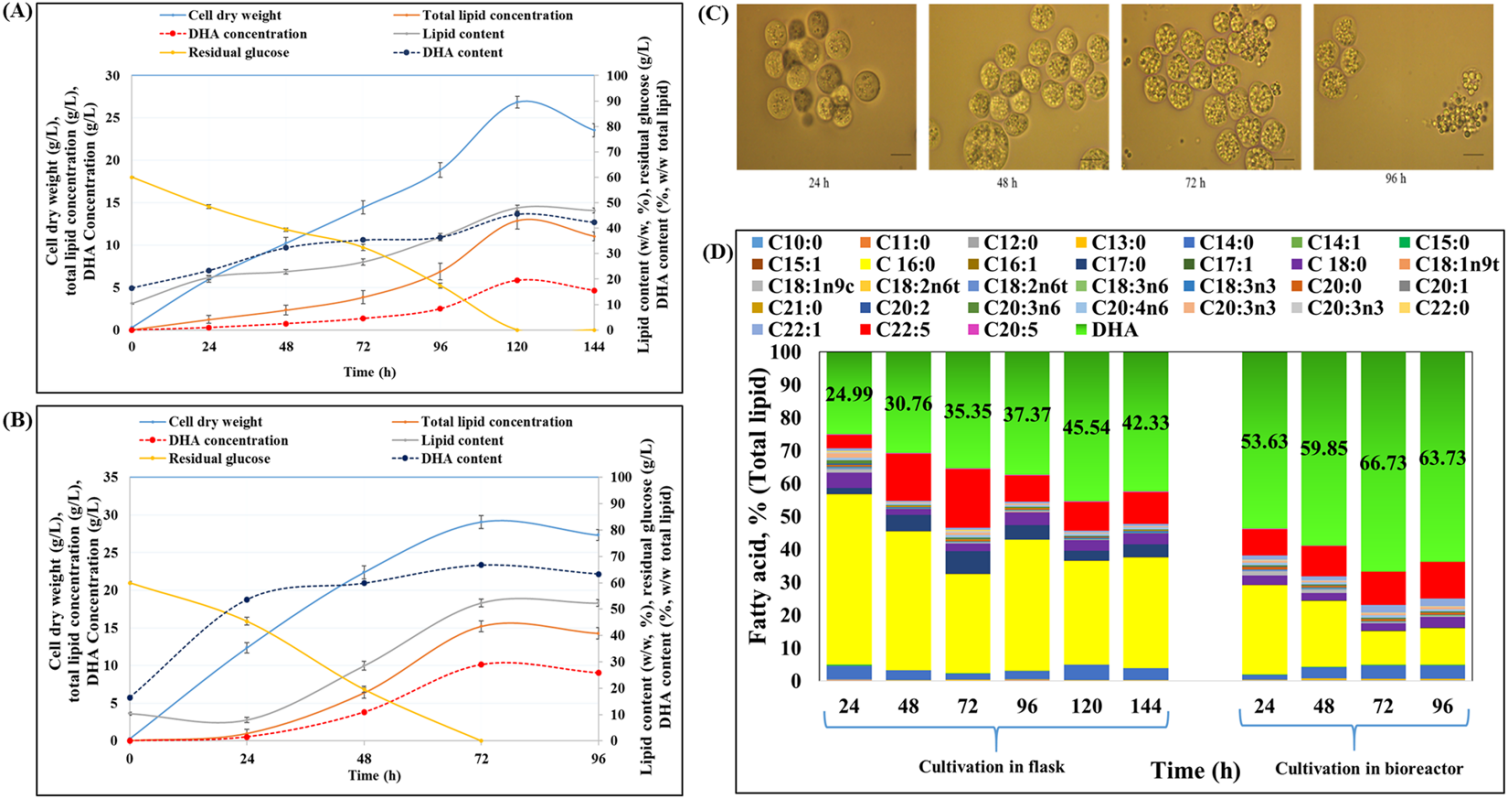
Time-course determination of cell dry weight, total lipid concentration, lipid content, residual glucose, DHA concentration, and DHA content of *S. limacinum* SR21 cultivated on OPSH containing 60 g/L of glucose in **(A)** Erlenmeyer flasks and **(B)** a bioreactor. **(C)** Morphological analysis of *S. limacinum* SR21 during cultivation on OPSH containing 60 g/L of glucose in a bioreactor. (D) Fatty acid profile (% w/w_total lipid_) of *S. limacinum* SR21 cultivated on OPSH containing 60 g/L of glucose in Erlenmeyer flasks and a bioreactor. Data values represent average with standard deviation.

Although previous studies have optimized the bioprocessing of DHA production, supplementation with glucose and glycerol obtained from renewable substrates has not been established until recently. Here, commercial glucose has been replaced with glucose derived from spruce biomass, which represents a renewable substrate. Spruce was treated with a hybrid organosolv-steam explosion method, that resulted into pretreated solids with high-level-cellulose-content (72% w/w)^57^. The solids (10% w/w) were further hydrolyzed by commercial enzymes that resulted into 64.70 g/L of glucose^57^. The concentration of glucose for the fermentation experiments was adjusted to 60 g/L with an appropriate amount of OPSH and growth occurred in flask and bioreactor (Fig. 4A,B). After consumption 11.57 g/L and 14.60 g/L of glucose from flask and bioreactor in 24 h, the cell dry weight were 5.98 ± 0.36 g/L and 12.36 ± 0.67 g/L, respectively, whereas the respective total lipid concentration were 1.23 ± 0.45 g/L and 0.98 ± 0.53 g/L. In the flask, cell dry weight was relatively linear between 24 and 96 h, but increased thereafter, driven by elevated lipid synthesis until all glucose was consumed (Fig. 4A). In a bioreactor, cell dry weight and lipid synthesis augmented linearly from 24 h until stationary phase at 72 h (Fig. 4B). In the initial phase of bioreactor growth (24 to 48 h), increasing cell numbers and growth contributed to the cell dry weight; thereafter, the cells stopped growing and started synthesizing lipids between 48 and 72 h (stationary phase) until all glucose was consumed (Fig. 4B). Accordingly, lipid-free biomass was 11.38 g/L at 24 h, increased to 16.01 g/L at 48 h, and declined to 13.86 g/L at 72 h. For *S. limacinum* SR21 cultivated in flasks, the highest cell dry weight (26.87 ± 0.69 g/L) and lipid concentration (12.87 ± 0.95 g/L) was observed at 120 h of cultivation with complete glucose utilization (Fig. 4A). While in the bioreactor, all glucose was consumed at 72 h of cultivation whereas the cell dry weight and total lipid concentrations were 29.07 ± 0.84 g/L and 15.21 ± 0.72 g/L, respectively that was corresponded to 52.32% (w/w) of lipid content (Fig. 4B). The high biomass and lipid accumulation in bioreactor was likely due to efficient oxygen transfer and continuous pH adjustment during cultivation^13^. Once all the C source was depleted, the cells started to consume their own lipids and a decrease in lipid concentration was observed after 72 h in the bioreactor and 144 h in the flask, phenomenon that is often observed in oleaginous microorganisms. Similarly, *Schizochytrium* sp. S31 grown in flask on 20 g/L of glucose showed 6.01 g/L of cell dry weight with 2.38 g/L of total lipid concentration after all sugar utilization in five days^58^. In the present study, *S. limacinum* SR21 displayed a sudden change in morphology at this stage, shifting from vegetative to reproductive growth (Fig. 4C). The pattern of lipid synthesis in this microorganism is like the other oleaginous microalgae e.g. *Crypthecodinium cohnii* and *Schizochytrium* sp. S31^59,60^.

In flask cultivation, the biomass and lipid productivity were 5.37 ± 0.23 g/L/d and 2.57 ± 0.25 g/L/d, respectively while with bioreactor cultivation, it was boosted to 9.69 ± 0.34 g/L/d and 5.07 ± 0.13 g/L/d, correspondingly (Table 1). Similarly, the biomass yield (0.48 ± 0.01 g/g_substrate_) and lipid yield (0.25 ± 0.01 g/g_substrate_) were observed higher in bioreactor cultivation at 72 h (Fig. 3A) than those obtained with flask cultivation at 120 h (0.44 ± 0.01 g/g_substrate_ and 0.21 ± 0.01 g/g_substrate,_ respectively).

**Table 1:** Summary of various parameters relating to cultivation of *Aurantiochytrium* sp. T66 on OPSH in flask and bioreactor. Data values represent average with standard deviation.

Parameters	Cultivation in flask	Cultivation in bioreactor
Cell dry weight (g/L)	26.87 ± 0.69	29.07 ± 0.84
Biomass yield (g/g_substrate_)	0.44 ± 0.01	0.48 ± 0.01
Biomass productivity (g/L/d)	5.37 ± 0.23	9.69 ± 0.34
Total lipid concentration (g/L)	12.87 ± 0.95	15.21 ± 0.72
Lipid content (%, w/w)	47.90 ± 1.23	52.32 ± 1.45
Lipid yield (g/g_substrate_)	0.21 ± 0.01	0.25 ± 0.01
Lipid productivity (g/L/d)	2.57 ± 0.25	5.07 ± 0.13
DHA content (%, w/w_total lipids_)	45.54 ± 0.23	66.72 ± 0.31
DHA concentration (g/L)	5.86 ± 0.34	10.15 ± 0.64
DHA yield (mg/g_CDW_)	218.08 ± 1.16	349.15 ± 1.54
DHA productivity (g/L/d)	1.17 ± 0.12	3.38 ± 0.27
Squalene yield (mg/g_CDW_)	16.34 ± 1.81	32.12 ± 4.37
Squalene concentration (mg/L)	439.05 ± 1.34	933.72 ± 6.53

Liang *et al*.^61^ reported 2.30 ± 0.06 g/L/d of biomass productivity and 1.15 ± 0.03 of lipid productivity, when *S. limacinum* SR21 was cultivated in flasks with 48 g/L of glucose^61^.

Overall, the fatty acid profile was similar to that of *S. limacinum* SR21 cultivated on pure glucose (Fig. 4D); the only difference was observed during extended cultivation after stationary phase. After 24 h, *S. limacinum* SR21 synthesized 24.99% DHA (w/w_total lipids_) when grown in a flask (Fig. 4A), but 53.63% w/w_total lipids_ in a bioreactor (Fig. 4B). The highest DHA content in a flask (46.36% w/w_total lipids_), corresponding to 5.86 g/L DHA, was observed after 120 h; whereas in a bioreactor, maximal DHA content (66.72% w/w_total lipids_) and concentration (10.14 g/L) were even higher and were achieved sooner (72 h) (Fig. 4B and Table 1). A list of DHA producing thraustochytrids is presented in Table 2.

**Table 2:** Production of DHA by marine thraustochytrid *S. limacinum* SR 21 on various substrates.

Microorganisms	Medium	Nitrogen source	Cell dry weight (g/L)	DHA content (%, total lipids)	DHA productivity (g/L/day)	References
*Schizochytrium* SR21	Glucose 60 g/L	Corn steep liquor 0.7 g	21.0	34.9	2.0	^51^
*S. limacinum* SR 21	Glucose:Glycerol (6:2) total carbon 9%	Yeast extract 1%	33.24	23.48	0.95^#^	^34^
	Glu:Gly (8:2) total carbon 9%		34.43	18.30	0.84^#^	
*A. limacinum* (ATCC MYA‐1381)	Gluocse 10 g/L	—	8.83	14.04^#^	0.99^#^	^30^
	Crude glycerol 10 g/L	—	8.86	15.01	1.06	
*S. limacicum* SR21	Glucose 48 g/L	—	10.90 ± 0.30	34	0.40 ± 0.05	^61^
	50% sorghum juice	—	9.38 ± 0.25	34	0.35 ± 0.02	
*Schizochytrium* sp.	1,500-L bioreactor using fed-batch fermentation (40 g/L glucose)	MSG	71	48.95	2.85^#^	^72^
*S. limacinum* SR21	Organosolv pretreated Spruce hydrolysate; OPSH (Flask)	Yeast extract (C/N; 10)	26.87 ± 0.69	45.54 ± 0.23	1.17 ± 0.12	This Study
	OPSH (Bioreactor)		29.07 ± 0.84	66.73 ± 0.31	3.38 ± 0.27	
	Glucose 60 g/L (Flask)		25.59 ± 0.32	46.38 ± 0.17	1.10 ± 0.16	
	Glucose 60 g/L (Bioreactor)		28.17 ± 0.78	67.76 ± 0.21	3.23 ± 0.31	

(-) not mentioned; # calculated from data.

**Figure 5:**
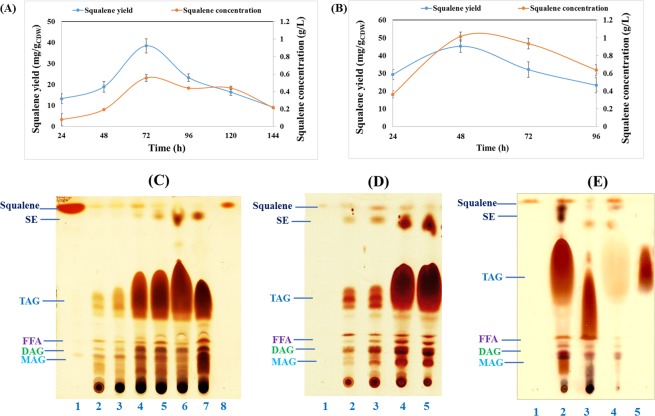
Evolution of squalene yield (mg/g_CDW_) and concentration (g/L) during cultivation of *S. limacinum* SR21 on OPSH containing 60 g/L of glucose in **(A)** Erlenmeyer flasks and **(B)** a bioreactor. **(C)** TLC analysis of extracted lipids from *S. limacinum* SR21. Analysis of squalene in total lipid extracts from *S. limacinum* SR21cultivated on OPSH containing 60 g/L of glucose in Erlenmeyer flasks where lane 1, squalene standard; lanes 2–7, samples cultivated for 24–144 h; lane 8, squalene standard. **(D)** *S. limacinum* SR21cultivated on OPSH containing 60 g/L of glucose in bioreactor where lane 1, squalene standard; lanes 2–5, samples cultivated for 24–96 h. **(E)** Purification of squalene from total lipid extracts following saponification: lane 1, squalene standard; lane 2, unsaponifiable fraction; lane 3, saponified fraction; lane 4, purified product from the unsaponifiable fraction; lane 5, triacylglycerol (TAG) standard. FFA, free fatty acids; DAG, diacylglycerol; MAG, monoacylglycerol. Images presented in Fig. 5C–E were obtained after TLC of different samples in different time frame.

The light orange color of thraustochytrids cultures is given by the presence of carotenoids, in which squalene, astaxanthin, canthaxanthin, and zeaxanthin are prominent carotenoids^13,62,63^. In the present study, squalene was extracted along with lipids from *S. limacinum* SR21 cultivated on OPSH in a flask and bioreactor (Fig. 5). Squalene yield increased from 13.23 mg/g_CDW_ (0.07 g/L) at 24 h to 38.45 mg/g_CDW_ (0.56 g/L) at 72 h of cultivation in the flask but dropped to 9.12 mg/g_CDW_ (0.21 g/L) at 144 h (Fig. 5A). This decline in squalene could be explained by the simultaneous formation of steryl ester (SE) from squalene, as identified by TLC (Fig. 5C, lanes 5, 6, 7). A similar trend was observed during bioreactor cultivation, whereby squalene yield increased from 29.32 mg/g_CDW_ (0.36 g/L) at 24 h to 45.38 mg/g_CDW_ (1.01 g/L) at 48 h, thus nearly doubling the values obtained with flask cultivation (Fig. 5B). The concentration then declined to 0.63 g/L at 96 h and a high amount of SE was observed at that point (Fig. 5D, lane 5). The bands for squalene from extracted lipid at 24 to 96 h was identified in Fig. 5D, lanes 2 to 5, where this squalene was purified as unsaponifiable fraction that was contaminated with some triacylglycerol and SE (Fig. 5E, lane 4) from saponified fraction of lipids with minor quantity of squalene (Fig. 5E, lane 3). The advanced purification of squalene can be performed by other chromatographic methods^25,64^. Nakazawa *et al*.^65^ used TLC to analyze almost 176 strains and identified 38 that were capable of synthesizing squalene^65^. Three different strains of *S. mangrovei* (FB1, FB2, FB3) were analyzed for squalene content, with FB1 reaching a maximum value of 0.162 mg/g_CDW_ (8.53 g/L CDW)^64^. In another study, addition of 0.1 mM methyl jasmonate to the cultivation medium increased squalene by 60% to 1.17 ± 0.006 mg/g_CDW_ after 48 h of cultivation^66^. In contrast to our results, *Aurantiochytrium* sp. 18W-13a synthesized high amounts of squalene (198 mg/g_CDW_) at 4 days of cultivation^67^.

## Conclusion

The present study optimized the cultivation parameters for attaining high cell dry weight and lipid accumulation by *S. limacinum* SR21 in flasks and a bioreactor. The highest biomass (0.39 g/gsubstrate) and lipid yields (0.20 g/gsubstrate) were obtained with 60 g/L glucose. The maximum DHA productivity with 60 g/L of pure glucose was 1.10 ± 0.16 g/L/d and 3.23 ± 0.31 g/L/d in flask and bioreactor cultivation, respectively. Pure glucose was replaced with organosolv-pretreated spruce hydrolysate (OPSH) to test the cost-effective production of nutraceuticals. In this case, biomass and lipid productivity were 9.69 ± 0.34 and 5.07 ± 0.13 g/L/d, respectively, when using a bioreactor. Similarly, bioreactor cultivation doubled the DHA content to 66.72 ± 0.31% w/w total lipids (10.15 g/L) and squalene content to 0.93 g/L, as compared to flask cultivation. The DHA productivity with OPSH containing 60 g/L glucose was 1.17 ± 0.12 g/L/d and 3.38 ± 0.27 g/L/d in flask and bioreactor cultivation, respectively. Importantly, we show that *S. limacinum* SR21 has great expertise to utilize as commercial strain for simultaneous production of DHA and squalene.

## Methods

### Microbial strain and cultivation conditions

The marine thraustochytrid *S. limacinum* SR21 (ATCC-MYA-1381) was procured from the American Type Culture Collection and was cultured in ATCC 790 By + medium containing yeast extract (1 g), peptone (1 g), and glucose (5 g) in artificial seawater (1000 mL). Medium pH was adjusted to 6.8 with 1 N HCl before sterilization. The content of artificial seawater was similar as reported in our previous studies^68^.

### Seed culture preparation

The seed culture of *S. limacinum* was cultivated in the above medium in 250-mL Erlenmeyer flasks with 100 mL working solution. The flasks were incubated for 48 h in an incubator at 25 °C with 180 rpm.

### Batch cultivation in Erlenmeyer flasks

Cultivation trials were carried out in Erlenmeyer flask (500-mL) with the 100 mL of working solution. To optimize the concentration of artificial seawater in the growth medium, the latter contained 30 g/L of glucose in 25%, 50%, 75%, 100% (v/v) artificial seawater adjusted with distilled water. Yeast extract was used as organic N source at a C/N (g/g) ratio of 10; this was based on the assumption that yeast extract represented 11.6% of total N with 6.2% amino N (Sigma-Aldrich, St. Louis, MO, USA). It has been already reported that the yeast extract (which contains 9–12% w/w of total N) obtained from brewery industries after processing with spent yeast biomass is utilized for cultivation of thraustochytrids^69^. To determine the effect of various C/N ratios (10, 20, 30, 40, 50), the appropriate amount of yeast extract was added together with 30 g/L of glucose. After selecting a suitable C/N ratio for maximum lipid production, various amounts of glucose (30, 60, 90, 120 g/L) were tested. To avoid Maillard’s reaction between amino acids in the yeast extract and reducing sugars, glucose was always autoclaved separately. Medium pH was adjusted to 6.8 with 1 N NaOH and 1 N HCl prior to autoclaving. After inoculation of medium with seed culture, the fermentation experiment was carried out in an incubator shaker with 180 rpm at 25 °C and sampling was done at every 24 h of cultivation for the analysis of growth and residual sugars.

### Batch cultivation in a bioreactor

Batch cultivation was carried out in a 3-L BioBundle bioreactor equipped with the ez2 control bundle (Applikon Biotechnology, JG Delft, The Netherlands) with 1 L working volume. The bioreactor was maintained at 25 °C and pH was adjusted with 3 N NaOH and 3 N HCl. Different aeration rates (0.66, 1.33, 2, 2.66 vvm) with fixed agitation speed at 300 rpm were tried to optimize biomass growth and lipid production by *S. limacinum*. To avoid excess moisture loss from the medium during cultivations with high aeration rate, a 5-L humidifier bottle was connected to the inlet air between air hose and cultivation vessel, and the condenser of the reactor was connected with an external thermostatic bath circulating with 90% glycol at 5 °C. After optimization of the aeration rate, a time-course experiment tested various amounts of glucose (30, 60, 90, 120 g/L) to study, by harvesting 50-mL samples every 24 h.

### Batch cultivation of *S. limacinum* with organosolv-pretreated spruce hydrolysate (OPSH)

Norway spruce chips obtained from mills in Northern Sweden was crushed into fine powder in a Retsch SM 300 knife mill (Retsch GmbH, Haan, Germany). The milled chips were subjected to pretreatment with hybrid organosolv-steam explosion method according to our optimized method^70^. The pretreated solids was obtained with high cellulose content (72%, w/w) including low hemicellulose (4%) and lignin (15.4%)^70^. The pretreated solids (10% of w/w) were hydrolyzed by commercial enzyme Cellic CTec2 (Novozymes A/S, Bagsværd, Denmark) at 20 FPU/g of solids in 50 mM citrate-phosphate buffer (pH 5) at 50 °C for 48 h.

The appropriate amount of hydrolysate was added to 50% v/v artificial seawater to adjust the glucose content at 60 g/L and finally, C/N ratio was maintained at 10 with yeast extract. The pH of medium was adjusted at 6.8. Cultivation experiments were carried out in Erlenmeyer flasks and bioreactor under optimized cultivation parameters as mentioned above.

### Analytical methods

Samples from Erlenmeyer flasks (15 mL) and bioreactor (50 mL) were aspirated off every 24 h to determine the growth, biomass, lipid accumulation and utilization of carbon source. The harvested samples were centrifuged at 8000 rpm for 10 min, and the supernatant was used for residual sugars analysis by HPLC equipped with Aminex HPX-87H column (Bio-Rad, Hercules, CA, USA) according to our established protocol^71^. The cell pellets were dried in a hot air oven at 40 °C till constant weight and the dry cell weight (DCW; g/L) was measured gravimetrically by weighing balance.

The oven-dried pellets were then pulverized into fine powder by using mortar and pestle and blended with a solution of chloroform and methanol (2:1, v/v). The slurry was transferred in screwed capped glass tubes followed by incubation under mild shaking at room temperature for 2 h. After that the slurry was mixed with ½ volume of water and allowed still for phase separation. The clear bottom layer of chloroform was aspirated in preweighed glass vials, the solvent was evaporated and the total lipids (g/L) were determined gravimetrically.

For the determination of squalene, the extracted lipids were dissolved in absolute acetonitrile and analyzed by HPLC (PerkinElmer, Waltham, MA, USA) equipped with C18 reverse-phase column (MACHEREY-NAGEL GmbH & Co. KG, Düren, Germany) using acetonitrile: water (9:1, v/v) as mobile phase with 1.5 mL/min flow rate at 30 °C. The quantification of squalene was done at UV detector at 210 nm with calibration curve prepared by standard solution of squalene with a range of 0.001 mg/mL to 10 mg/mL (Sigma-Aldrich, St. Louis, MO, USA). The extraction of squalene from total lipid was carried out by saponification method mentioned by Nakazawa *et al*.^65^. The squalene was separated as unsaponifiable fraction from the saponifiable lipid fraction. The purity of both fractions was analyzed by thin-layer chromatography (TLC). The fractions were spotted on silica gel 60 F_254_ normal phase plates (Merk, Dermstadt, Germany) and chromatogram was developed by using of *n-*hexane: diethyl ether: acetic acid (85:15:1; v/v/v) as mobile phase. The after spraying the methanolic MnCl_2_ solution (MnCl_2_.4H_2_O, 0.32 g; water, 30 mL; methanol, 30 mL, and H_2_SO_4_, 4 mL). After drying the plates in air, spots were visualized by charring of plates in hot air oven at 125 °C for 5 min. Triolein and pure squalene were used as standard.

The total lipids were transesterified for the estimation of fatty acids profile by using acid catalysts as described previously^68^. The fatty acid methyl esters (FAMEs) were estimated by GC-FID (Agilent, Santa Clara, CA, USA) equipped with capillary column Select FAME; dimensions 50 m × 0.25 mm ID and 0.25 μm film thickness. The quantification of individual peaks was carried out by using standard mixture of FAME, Supelco 37 Component FAME Mix (47885-U, Sigma-Aldrich).

### Statistical analysis

All experiments were carried out in triplicates to achieve the average value and errors bars by ± standard deviation. One-way analysis of variance (ANOVA) with p < 0.05 for data acceptance was performed with Microsoft Office Excel 2016 (Microsoft, USA).

## Supplementary information

Supplementary Material.
