# Supplementary material for: Co-production of DHA and squalene by thraustochytrid from forest biomass
Source: Sci Rep. 2020 Feb 6;10:1992. doi: 10.1038/s41598-020-58728-7 (PMC7005032; doi:10.1038/s41598-020-58728-7)

**Co-production of DHA and squalene by thraustochytrid from forest biomass**

Alok Patel, Stephan Liefeldt, Ulrika Rova, Paul Christakopoulos, Leonidas Matsakas*

Biochemical Process Engineering, Division of Chemical Engineering, Department of Civil, Environmental, and Natural Resources Engineering, Luleå University of Technology, SE-971 87 Luleå, Sweden

Alok Patel, email: alok.kumar.patel@ltu.se

Stephan Liefeldt: stephan.liefeldt@ltu.se

Ulrika Rova, email: ulrika.rova@ltu.se, Tel: +46 92 049 13 15

Paul Christakopoulos, email: paul.christakopoulos@ltu.se, Tel: +46 92 049 25 10

*Corresponding author: Leonidas Matsakas, Department of Civil, Environmental and Natural Resources Engineering, SE-971 87 Luleå Sweden, leonidas.matsakas@ltu.se, tel.: +46 (0) 920 493043

**Supplementary Fig. S1.** Representative microscopic image of *S. limacinum* SR21 cultivated on 60 g/L of glucose with two different C/N ratio (C/N 10 and C/N 50), where it showed the formation of zoosporangia (green arrow) as a result of sexual reproduction at high C/N ratio (C/N 50) and the emptied cells (red arrow) are clearly visualized after releasing of zoospores from the cells.


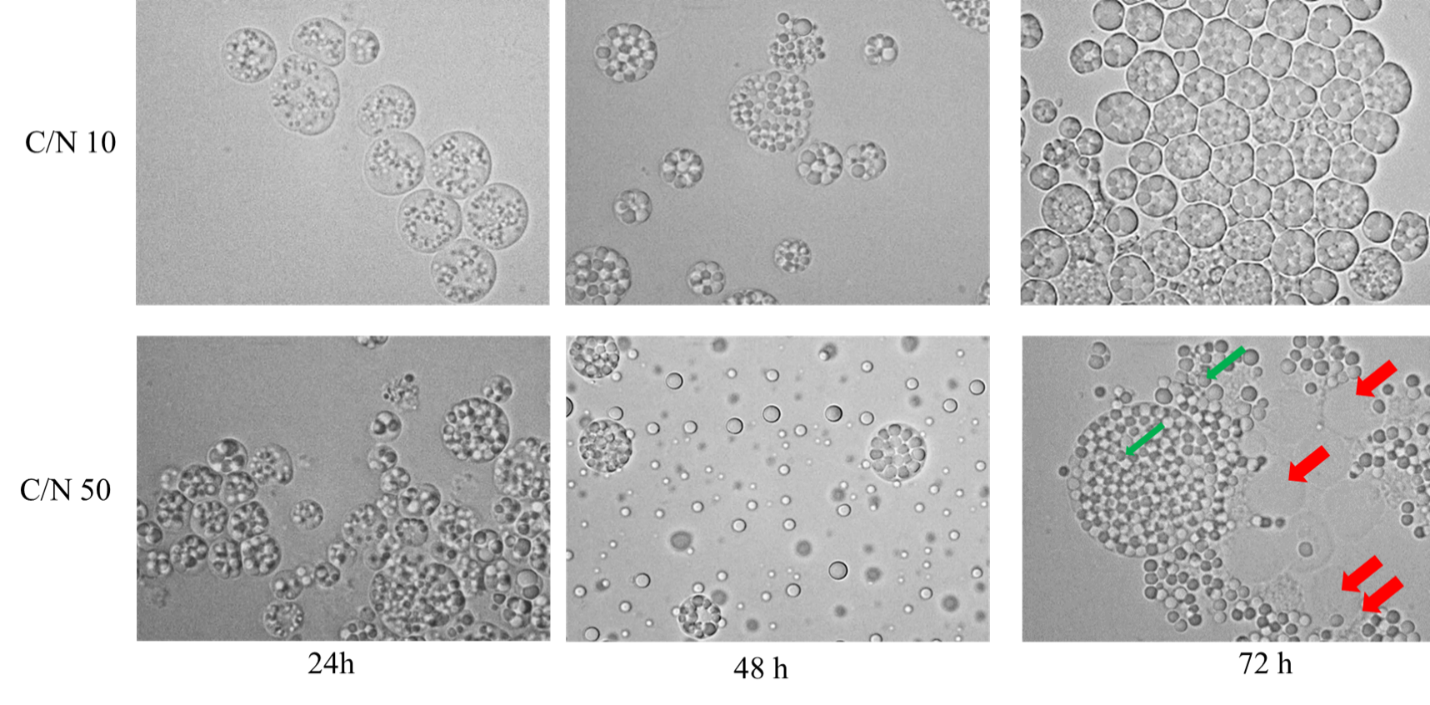


**Supplementary Fig. S2.** Time-course determination of cell dry weight (g/L), total lipid concentration (g/L), lipid content (%, w/w), and residual glucose (g/L) of S. limacinum SR21cultivated in Erlenmeyer (A-D): **(A)** 30 g/L glucose; **(B)** 60 g/L glucose; **(C)** 90 g/L glucose; **(D)** 120 g/L glucose flasks. Time-course determination of cell dry weight (g/L), total lipid concentration (g/L), lipid content (%, w/w), and residual glucose (g/L) of S. limacinum SR21cultivated in a bioreactor (E-H): **(E)** 30 g/L glucose; **(F)** 60 g/L glucose; **(G)** 90 g/L glucose; **(H)** 120 g/L glucose.


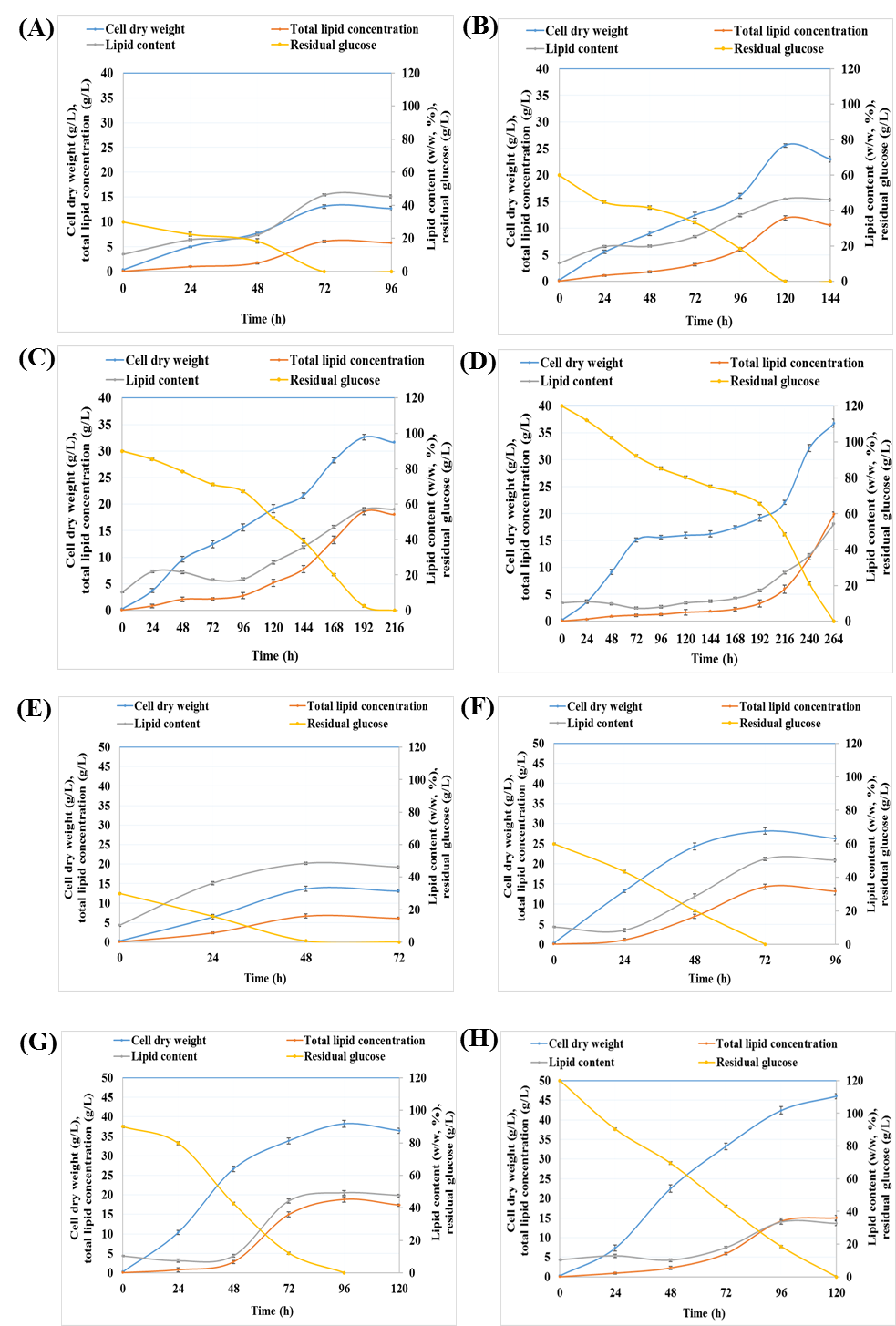

Supplement: Supplementary file 1 — Supplementary Material. [file 41598_2020_58728_MOESM1_ESM.docx]
